# Flow Cytometric Assessment of Sperm DNA Fragmentation by TUNEL and Acridine Orange: Methodological and Clinical Insights

**DOI:** 10.3390/jcm15020403

**Published:** 2026-01-06

**Authors:** Mohamed Abdelkarim, Nadine Ghannem, Khadija Kacem-Berjeb, Sana Chtourou, Linda Debbabi, Anis Fadhlaoui, Mounir Ben Mefeteh, Fethi Zhioua, Marouen Braham, Nozha Chakroun

**Affiliations:** 1 Human Genetics Laboratory, Faculty of Medicine of Tunis, Tunis El Manar University, Tunis 1006, Tunisia; mohamed.abdelkarim@fmt.utm.tn (M.A.);; 2 Department of Reproductive Biology and Cytogenetics, Aziza Othmana Hospital, Tunis El Manar University, Tunis 1008, Tunisiasana_cht@yahoo.com (S.C.);; 3 Research Laboratory LR16SP01 “Infertility and Oncofertility”, Tunis 1006, Tunisia; 4 Gynecology and Obstetrics Department, Aziza Othmana Hospital, Tunis 1008, Tunisia

**Keywords:** male infertility, sperm DNA fragmentation, TUNEL, acridine orange, flow cytometry

## Abstract

**Background/Objectives:** Male infertility contributes to nearly half of global infertility cases, yet conventional semen analysis is insufficient to predict assisted reproductive technology (ART) outcomes such as intracytoplasmic sperm injection (ICSI). Sperm DNA fragmentation (SDF) is a promising biomarker of genomic integrity, but clinical implementation remains hindered by methodological heterogeneity. This study compared two SDF assays—TUNEL and Acridine Orange (AO)—regarding their correlations with semen parameters and ICSI outcomes. **Methods:** Sixty men undergoing ICSI were prospectively enrolled. SDF was analyzed using two flow cytometric assays: TUNEL (detecting DNA strand breaks) and AO (assessing chromatin instability). Semen quality and ICSI outcomes (fertilization, cleavage, blastulation, and embryo utilization rates) were evaluated. Statistical analyses included Spearman’s rank correlation and Mann–Whitney tests. **Results:** Median SDF levels were significantly higher by TUNEL than AO (17.2% vs. 10.15%; *p* = 0.0065). Inter-assay correlation was weak (*r* = 0.299, *p* = 0.01). AO-derived SDF correlated positively with age (*r* = 0.311, *p* = 0.02), while TUNEL showed no such trend. Neither assay correlated significantly with semen parameters or ICSI outcomes, although AO tended to associate with lower motility and slightly reduced top-quality embryo rates. **Conclusions:** TUNEL and AO capture distinct facets of sperm DNA damage. Their limited correlation and lack of predictive value for ICSI outcomes highlight the need for assay-specific interpretation and standardization. Integration of SDF with additional biomarkers and oocyte factors may enhance its clinical utility.

## 1. Introduction

Male infertility accounts for approximately 40–50 percent of infertility cases globally [1]. Despite this significant prevalence, conventional semen analysis—predicated on parameters such as sperm concentration, motility, and morphology—often proves inadequate for predicting reproductive success, particularly in the context of assisted reproductive technologies (ART) such as intracytoplasmic sperm injection (ICSI) [2]. This diagnostic gap has catalyzed interest in the identification of advanced biomarkers of sperm quality, among which sperm DNA fragmentation (SDF) has emerged as a critical predictor of genetic integrity and embryogenic potential [3,4]. Nevertheless, the clinical utility of SDF testing is hindered by methodological variability, with different assays measuring distinct aspects of DNA damage and chromatin anomalies [5]. In addition to the Acridine Orange–based approach, the Sperm Chromatin Structure Assay (SCSA) is one of the most widely used and best standardized methods for assessing sperm DNA fragmentation. SCSA evaluates chromatin susceptibility to acid-induced denaturation using acridine orange fluorescence and provides quantitative parameters such as the DNA Fragmentation Index (DFI) and High DNA Stainability (HDS). It is considered the most standardized SDF test and is recommended by the WHO for both clinical and research applications.

Several techniques have been developed to assess sperm DNA integrity, including the Terminal deoxynucleotidyl transferase dUTP Nick-End Labeling (TUNEL) assay and Acridine Orange (AO) staining, both of which can be adapted to flow cytometry [6]. At this stage, it is important to specify that both the TUNEL assay and the SCSA are currently regarded as standardized and WHO-recommended tests for sperm DNA fragmentation. Although based on acridine orange fluorescence, the SCSA represents a validated flow cytometric approach that differs substantially from the older microscopic AO test, which is considered unreliable for clinical use. The present study refers exclusively to the flow cytometric AO method derived from SCSA principles, rather than the microscopic variant. The TUNEL assay quantifies DNA strand breaks by enzymatic labeling, whereas AO staining assesses chromatin stability through metachromatic fluorescence shifts between double- and single-stranded DNA. Among flow cytometric methods, the Acridine Orange assay relies on the same biophysical principle as the Sperm Chromatin Structure Assay (SCSA), as both evaluate DNA denaturation susceptibility using metachromatic fluorescence. These methods measure distinct, yet complementary, aspects of DNA damage: TUNEL identifies actual strand breaks, while AO reflects chromatin compaction and denaturation susceptibility and may consequently indicate various forms of sperm DNA damage [7,8,9,10].

Previous studies have produced mixed results regarding the comparability and predictive value of these assays. For instance, Henkel et al. [11] and Hamidi et al. [12] demonstrated that both TUNEL and AO can identify high SDF associated with reduced fertilization and pregnancy rates in IVF/ICSI cycles, though AO—when used in its SCSA-derived, flow cytometric form—sometimes correlates better with sperm motility as it reflects chromatin susceptibility to acid-induced denaturation rather than true DNA strand breaks. Conversely, other reports, such as Sun et al. [13], found no significant relationship between SDF levels and ART outcomes, highlighting the ongoing debate about assay interpretation thresholds. A recent meta-analysis encompassing over 25,000 ART cycles further confirmed that methodological choice significantly affects both detection rates and clinical conclusions [14].

Given this context, the present study aims to compare the TUNEL assay with the flow cytometric AO method—an assay derived from the SCSA principles but distinct from the standardized SCSA protocol—for evaluating sperm DNA fragmentation within a well-characterized ICSI cohort. Specifically, we examined their respective correlations with semen parameters and embryological outcomes and evaluated whether assay-specific thresholds influence clinical interpretation. By clarifying methodological differences and their potential clinical implications, this work seeks to contribute to the standardization of SDF testing in ART laboratories and improve the assessment of male reproductive potential.

## 2. Materials and Methods

### 2.1. Chemicals

The following reagents were used for the preparation of solutions: NaCl (Sigma-Aldrich, D-3016, St. Louis, MO, USA), Tris (Sigma-Aldrich, T1503, St. Louis, MO, USA), EDTA (Sigma-Aldrich, 28021, St. Louis, MO, USA), Triton X-100 (Merck KGaA, 52H0286, Darmstadt, Germany), Acridine Orange (Sigma-Aldrich, 10127-02-3, St. Louis, MO, USA), citric acid (Sigma-Aldrich, 27781, St. Louis, MO, USA), Na_2_HPO_4_ (Sigma-Aldrich, A836486, St. Louis, MO, USA), paraformaldehyde (VWR, 9650819, Radnor, PA, USA), and phosphate-buffered saline (Eurobio Scientific, CP21-4453, Les Ulis, France).

### 2.2. Patients

This prospective study was conducted from April to November 2023 at the Laboratory of Biology and Medically Assisted Procreation, Aziza Othmana University Hospital, Tunis. Sixty men undergoing ICSI were included. This study was conducted at Aziza Othmena Hospital. The study protocol was approved by the local ethics committee of Aziza Othmena Hospital (Approval No. 07-2023). Approval date: 11 March 2023. Informed consent was obtained from all subjects involved in the study.

Inclusion criteria required collection of sperm samples on the same day as ICSI to ensure fresh sample analysis.

**Figure 1:**
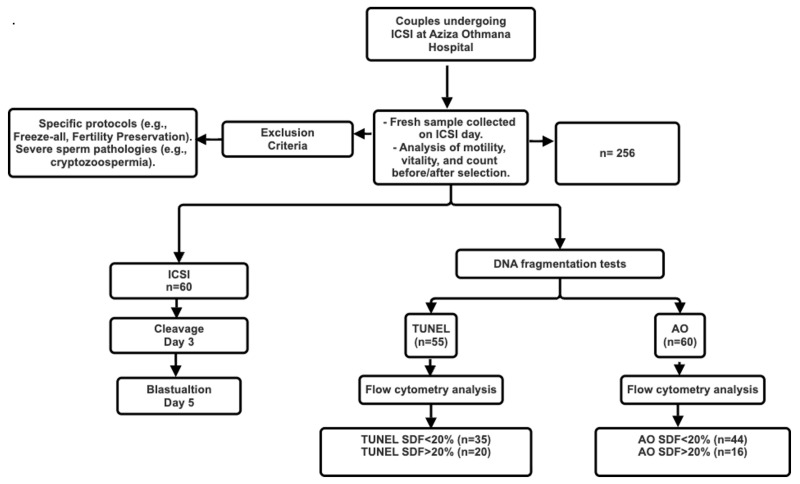
Flow Chart of Sperm DNA Fragmentation Analysis in ICSI Patients: From Sample Collection to Embryo Development and Pregnancy Outcome.

Exclusion criteria included cycles using the Mild Protocol, Freeze-all, Fertility Preservation, or ICSI with testicular or frozen spermatozoa, as well as patients with cryptozoospermia, hypospermia, or extreme oligoasthenoteratozoospermia (Figure 1).

All participants underwent a standardized clinical interview and semen analysis following World Health Organization guidelines.

### 2.3. Semen Collection and Analysis

Semen samples were collected by masturbation after 2–5 days of sexual abstinence and allowed to liquefy at 37 °C for 30 min. Semen volume was measured using a 5 mL pipette, and sperm motility was evaluated within 1 h under phase-contrast microscopy.

Sperm preparation was performed using density-gradient centrifugation with SpermGrad™ (Vitrolife, Göteborg, Sweden) and GIVF™PLUS media (Vitrolife, Denver, CO, USA) at 90% and 45%, respectively. After centrifugation and washing, motile spermatozoa were recovered and analyzed for concentration, motility, morphology, and DNA fragmentation.

### 2.4. Intracytoplasmic Sperm Injection (ICSI)

Oocytes were assessed for maturity, and morphologically normal motile sperm were selected for microinjection. Fertilization was confirmed by the presence of two pronuclei (2PN), and embryo development was monitored through the cleavage and blastulation stages.

The following ICSI outcomes were recorded: fertilization rate (2PN/Metaphase II oocytes), cleavage rate (Day 2–3 embryos/2PN), top-quality embryo rate (top-quality embryos/total embryos), blastulation rate (blastocysts/total embryos cultured), blastocyst utilization rate (usable blastocysts/total blastocysts), and pregnancy rate (positive β-hCG/embryo transfers).

### 2.5. TUNEL Assay

Sperm DNA fragmentation was assessed using the APO-DIRECT™ kit (BD Biosciences, 556381, San Jose, CA, USA) according to the manufacturer’s protocol.

Briefly, sperm samples were washed, centrifuged, and resuspended in 3% paraformaldehyde, followed by fixation in 70% ethanol and storage at −20 °C. Fixed spermatozoa were labeled using terminal deoxynucleotidyl transferase (TdT) and fluorescein-dUTP. After washing, samples were counterstained with propidium iodide (PI) to visualize DNA content.

Flow cytometric analysis was performed using a BD FACS Canto II cytometer, and data were analyzed with BD FACS Diva 7 software. Results were expressed as the percentage of spermatozoa with fragmented DNA, using the provided positive and negative controls.

### 2.6. AO Assay (SCSA-Derived)

AO staining followed the modified Sperm Chromatin Structure Assay (SCSA) protocol described by Evenson and Jost [15].

Aliquots were diluted in TNE buffer (0.15 mol/L NaCl, 0.1 mol/L Tris, 1 mmol/L EDTA, pH 7.4) and stored at −70 °C. Before analysis, samples were thawed and adjusted to 2 × 10^6^ sperm/mL in TNE. After 30 s of acid-detergent treatment (0.1% Triton X-100, 0.15 mol/L NaCl, 0.08 N HCl, pH 1.2), spermatozoa were stained with AO (6 µg/mL).

Fluorescence was recorded for 10,000 events per sample using a BD FACS Canto II flow cytometer: green fluorescence (double-stranded DNA) was detected at 530 nm (band-pass filter) and red fluorescence (single-stranded DNA) at 650 nm (long-pass filter). Data were analyzed with BD FACS Diva 7 software, and SDF (%) was calculated as the proportion of cells exhibiting red fluorescence. The detailed flow cytometry gating strategy and representative fluorescence dot plots used to identify the sperm population and determine MFD are provided in Supplementary Figure S1.

### 2.7. Statistical Analysis

Statistical analyses were performed using GraphPad InStat 5 (GraphPad Software Inc., San Diego, CA, USA). Data normality was evaluated using the Shapiro–Wilk test. Continuous variables are expressed as mean ± standard deviation (SD) and median (interquartile range).

Group comparisons were performed using the Mann–Whitney U test for non-parametric data. Spearman’s rank correlation coefficient was used to assess associations between SDF and clinical parameters. A *p* < 0.05 was considered statistically significant.

## 3. Results

The study cohort included 60 men with a mean age of 40.77 ± 5.1 years. The mean sperm concentration was 82.7 ± 98.69 M/mL, and the mean progressive motility was 32.31 ± 16.4%. Regarding ICSI outcomes, the mean fertilization rate was 65.6 ± 30.6%, the cleavage rate was 84.83 ± 29.82%, and the blastocyst utilization rate was 15.44 ± 34.8%. Detailed demographic, semen, and embryological data are presented in Table 1.

### 3.1. Comparison Between TUNEL and Acridine Orange Assays

Sperm DNA fragmentation (SDF) values obtained with TUNEL and Acridine Orange (AO) assays showed a weak but statistically significant correlation (*r* = 0.299, *p* = 0.01).

The median SDF level determined by the TUNEL assay (17.20%) was significantly higher than that obtained with AO (10.15%, *p* = 0.0065). These results illustrate the distinct biological aspects captured by the two flow cytometric approaches (Table 2).

This table summarizes the median values (with interquartile ranges) of DNA fragmentation percentages measured by TUNEL and AO assays in a cohort of 60 individuals. A statistically significant difference was observed between the two methods (*p* < 0.05). Furthermore, Spearman’s rank correlation analysis revealed a significant positive correlation between TUNEL and AO results (*r* = 0.299, *p* < 0.05).

### 3.2. Correlations Between SDF and Clinical Parameters

A weak but significant positive correlation between age and SDF was observed using the AO method (*r* = 0.311, *p* = 0.02), whereas no association was found with TUNEL (*r* = 0.07, *p* = 0.59).

Neither assay showed significant correlations with sperm concentration (TUNEL: *r* = 0.029, *p* = 0.842; AO: *r* = −0.004, *p* = 0.975) or progressive motility.

In relation to ICSI outcomes, no significant associations were observed between SDF values (from either assay) and fertilization, cleavage, or blastulation rates. However, a moderate but non-significant positive trend was observed between AO-derived SDF and the blastocyst utilization rate (*r* = 0.267, *p* = 0.139) (Table 3).

### 3.3. Stratification by SDF Threshold (<20% vs. >20%)

The 20% cut-off used to distinguish low- and high-SDF groups was selected based on previously published thresholds commonly applied in studies using both TUNEL and AO/SCSA-derived DNA fragmentation. Several clinical and meta-analytic reports have consistently applied thresholds around 20% to define clinically relevant levels of sperm DNA damage in ART populations [16]. To ensure methodological coherence and enable comparison with earlier research, the present study adopted the same threshold. Importantly, this value was used solely for analytical subgrouping and does not represent a diagnostic or clinically validated cut-off. This approach also aligns with threshold ranges frequently reported in the literature, typically 15–20% for TUNEL and 20–25% for AO/SCSA. To further explore potential assay-specific effects, patients were classified into low-SDF (<20%) and high-SDF (>20%) groups.

Among the 60 participants, 31.7% had SDF > 20% according to TUNEL, and 28.3% according to AO.

Within the low-SDF group, no significant correlations were observed between SDF and age, semen parameters, or ICSI outcomes. AO showed a negative trend with progressive motility (*r* = −0.397, *p* = 0.127) and a borderline positive association with top-quality embryo rate (*r* = 0.485, *p* = 0.07). In the high-SDF group, both assays displayed non-significant correlations with the evaluated parameters, suggesting that variations in sperm DNA damage above 20% did not consistently influence clinical outcomes (Table 4).

### 3.4. Summary of Findings

Overall, TUNEL produced higher SDF values than AO. Age showed a modest positive correlation with AO-derived SDF, whereas no such correlation was observed with TUNEL. In this cohort, no statistically significant relationships were detected between SDF values and semen parameters or ICSI outcomes; however, these findings should be interpreted cautiously given the limited sample size.

While non-significant, the mild trends observed between AO-derived SDF and both blastocyst utilization and top-quality embryo rates suggest potential biological relevance that may warrant further investigation in larger cohorts.

## 4. Discussion

Sperm DNA damage comprises a wide spectrum of molecular alterations, including single-strand breaks (SSBs) and double-strand breaks (DSBs), which may result from oxidative stress, abnormal chromatin remodeling, or defective DNA–protein crosslinking [17]. These lesions compromise genome integrity and can affect fertilization, embryonic development, and even transgenerational inheritance. To detect them, a variety of assays have been developed, each characterized by specific biochemical targets and detection mechanisms.

**Figure 2:**
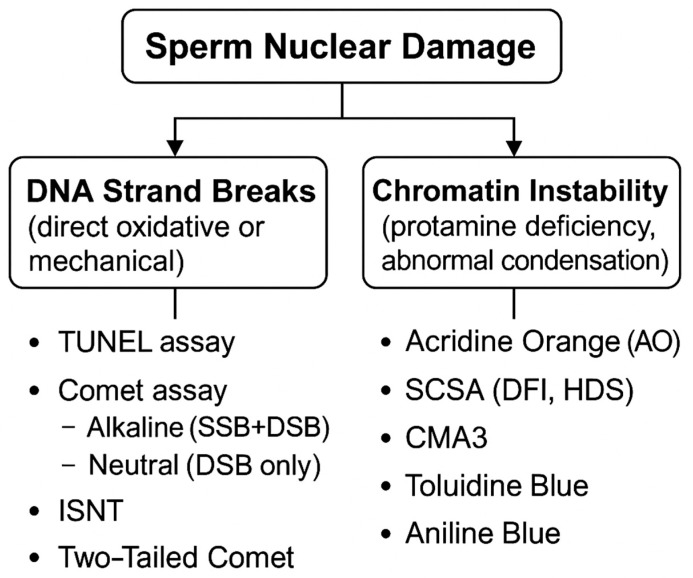
Overview of DNA Damage Types and Detection Methods. SSB: Single-Strand Break, DSB: Double-Strand Break, DFI: DNA Fragmentation Index, HDS: High DNA Stainability, SCSA: Sperm Chromatin Structure Assay.

Among the most frequently used SDF tests are the TUNEL assay, the SCSA, and the Halosperm (SCD) test, which are currently the most widely adopted methods in clinical andrology laboratories [17,18,19]. The alkaline comet assay detects both SSBs and DSBs, whereas the neutral comet assay is specific for DSBs. Fluorescent dyes such as Chromomycin A3, Toluidine Blue, and Aniline Blue provide information on chromatin condensation and protamine deficiency, whereas Acridine Orange (AO) evaluates DNA denaturation susceptibility and not true DNA damage [20]. The advent of flow cytometry has made it possible to analyze thousands of sperm cells rapidly and reproducibly, increasing the clinical relevance of these techniques. Figure 2 summarizes the principles of the major sperm DNA integrity assays.

Although the present study focuses on TUNEL and Acridine Orange assays, it is important to note that the SCSA is regarded as the most standardized and clinically validated SDF test, with WHO recognition. The AO protocol used in our study is adapted from the original SCSA method, sharing the same biophysical principle based on the metachromatic shift of acridine orange. However, unlike SCSA, which provides DFI and HDS as standardized outputs, our AO approach offers a simplified fluorescence-based estimation of chromatin instability. This distinction has now been clarified to contextualize our methodology in relation to the broader spectrum of SDF testing. The weak but significant correlation observed between TUNEL and AO in our study indicates that these two tests evaluate related yet distinct molecular phenomena. TUNEL directly labels 3′-OH termini of fragmented DNA using terminal deoxynucleotidyl transferase (TdT), thereby detecting strand breaks produced by oxidative stress, apoptosis, or enzymatic cleavage during defective spermiogenesis [21,22]. In contrast, AO detects the metachromatic shift in fluorescence that occurs when the dye intercalates into double-stranded (green) or single-stranded (red) DNA regions [23]. Therefore, while TUNEL quantifies actual DNA fragmentation, AO primarily reflects chromatin compaction status and the susceptibility of DNA to denaturation—parameters associated with protamine deficiency and altered histone–protamine transition [24,25,26]. The present work focused exclusively on two flow cytometric assays, TUNEL and AO, and did not include the complete SCSA protocol with its standardized gating strategy. This decision reflects the initial methodological scope established for the study and the laboratory resources available at the time. Nevertheless, Section 4 has been expanded to provide a more balanced comparison between the assays used in this study and the SCSA method, highlighting their respective strengths, limitations, and the additional level of standardization and analytical robustness offered by SCSA.

The higher SDF percentages obtained by TUNEL compared with AO confirm the greater sensitivity of TUNEL to irreversible strand breaks, particularly DSBs that are unlikely to be repaired post-fertilization [27,28,29]. Such breaks are known to hinder pronuclear formation, reduce embryo cleavage, and increase early embryonic arrest. Conversely, AO, which primarily reflects chromatin structural integrity, yielded lower SDF values. Although such chromatin anomalies may not impede fertilization per se, they could interfere with epigenetic reprogramming and gene expression during blastulation and implantation [30,31].

Interestingly, in our cohort, AO-derived SDF showed a modest positive correlation with age, whereas TUNEL did not. This suggests that age-related oxidative processes progressively weaken chromatin condensation without necessarily inducing physical strand breaks [32]. Previous reports have demonstrated that aging is accompanied by protamine depletion, increased histone retention, and accumulation of reactive oxygen species, all of which impair chromatin compaction and increase DNA susceptibility to denaturation [33].

In our cohort, no correlation was observed between SDF and sperm concentration. This finding is consistent with Cohen-Bacrie et al. [34], although other studies have reported differing results, highlighting that this association remains variable across reports [35]. This reinforces the notion that DNA fragmentation reflects sperm nuclear quality rather than overall sperm count or morphology. In our cohort, no significant relationships were observed between SDF and ICSI outcomes. While some studies suggest that oocytes may repair moderate sperm DNA lesions [36], numerous reports using TUNEL, SCSA or other assays have demonstrated associations between elevated SDF and reduced ART success, highlighting that the impact of SDF on clinical outcomes remains heterogeneous across studies. Such oocyte repair mechanisms may contribute to the absence of associations with certain early ICSI parameters in some studies, although this remains debated, as other reports have shown reduced fertilization or cleavage rates in cases of high SDF.

Nevertheless, our subgroup analyses revealed non-significant tendencies that may be of biological interest; however, these observations should be interpreted with caution given the small sample sizes. In the low-SDF group (<20%), AO results showed a negative trend with progressive motility (*r* = −0.397, *p* = 0.127) and a positive trend with the proportion of top-quality embryos (*r* = 0.485, *p* = 0.07). These findings may indicate that chromatin instability compromises mitochondrial function or axonemal structure, both of which are critical for sperm motility [37,38,39]. However, embryos derived from sperm with moderate chromatin defects may undergo selective attrition, leaving only the most viable embryos for transfer—a form of natural selection at the embryonic level.

In our cohort, no relationship was found between SDF and blastocyst utilization rate. While this observation aligns with reports indicating that blastocyst viability depends predominantly on ooplasmic competence rather than sperm genomic integrity [13,40,41,42], other studies have reported significant associations between elevated SDF and reduced blastulation or blastocyst quality, suggesting that this relationship remains inconsistent across the literature. Oocytes with sufficient repair capacity can correct a substantial portion of sperm DNA damage during early embryogenesis, though the potential long-term effects on implantation or fetal development remain uncertain.

Taken together, these data indicate that SDF reflects a complex interplay between oxidative stress, chromatin packaging, and the efficiency of oocyte-mediated DNA repair. The modest correlation between TUNEL and AO demonstrates that no single test can fully capture the spectrum of sperm genomic instability. Each assay provides complementary insights: TUNEL detects irreversible strand breaks, while AO reveals chromatin vulnerability. Their combined use, together with functional assays assessing oxidative stress or sperm membrane integrity, may offer a more comprehensive picture of sperm quality in ART settings.

Despite the value of these findings, some limitations should be acknowledged. The sample size was moderate, particularly in the high-SDF subgroups, which may limit statistical power. Female factors—such as oocyte age, quality, and repair capacity—were not included, yet these are known to modulate the impact of sperm DNA damage on ART outcomes. Another methodological limitation is the heterogeneity among patient characteristics, including male age, oocyte quality, and other reproductive factors that were not controlled for in this study. These variables are known to strongly influence fertilization, embryo development, and implantation, and their variability may reduce the ability to detect associations specifically attributable to sperm DNA fragmentation. This heterogeneity should be considered when interpreting the predictive relevance of the findings. In addition, our study was restricted to flow cytometric methods; integrating results from other assays, such as SCSA or comet analysis, could provide a broader comparative framework. The subcategorization into low- and high-SDF groups inevitably reduced the number of participants within each category, which limits the statistical power of subgroup analyses. These results should therefore be interpreted with caution, as smaller sample sizes may not allow subtle associations to emerge. Additional published studies supporting or contrasting these trends have now been integrated to contextualize the present findings within the broader literature. Future multicenter studies should aim to harmonize protocols and define clinically meaningful SDF thresholds to guide clinical decision-making in male infertility.

## 5. Conclusions

This study provides an in-depth comparison of two flow cytometric assays—TUNEL and Acridine Orange—for evaluating sperm DNA fragmentation. Although the methods correlated weakly, TUNEL detected higher SDF levels, likely reflecting true DNA strand breaks, while AO identified chromatin instability related to protamine deficiency. Neither assay independently predicted ICSI outcomes, indicating that SDF should be interpreted in conjunction with semen parameters and oocyte competence.

Standardization of SDF measurement protocols and integration with additional biomarkers, such as oxidative stress and sperm functional assays, are essential to enhance the clinical applicability of DNA fragmentation testing in male infertility. Combined approaches may ultimately help refine patient selection for ART and improve individualized therapeutic strategies.

## Figures and Tables

**Table 1:** Characteristics of Study Population.

	Mean ± Standard Deviation	Median (IQR)
Age (years)		
Male	40.77 ± 5.1	
Sperm parameters		
Sperm concentration (M/mL)	82.7 ± 98.69	55.0 (18.5–115.0)
Progressive motility (%)	32.31 ± 16.4	30 (20–40)
ICSI Results		
Fertilization Rate (%)	65.6 ± 30.6	
Cleavage Rate (%)	84.83 ± 29.82	
Number of Embryos (%)	3.26 ± 2.59	
Number of Top Embryos (Day 2) (%)	0.98 ± 1.62	
Top Embryos Rate (%)	28.15 ± 36.73	
Number of Blastocysts (%)	0.50 ± 1.04	
Blastulation Rate (%)	12.21 ± 23.55	
Blastocyst Utilization Rate (%)	15.44 ± 34.8	

**Table 2:** Comparison of TUNEL and Acridine Orange Assays for Sperm DNA Fragmentation Detection.

	Median (First Quartile, Third Quartile)	Spearman’s Rank Correlation (TUNEL vs. AO)
SDF		*r* = 0.299, *p <* 0.05
TUNEL (%)	17.20 (10.40–27.10)	
AO (%)	10.15 (6.40–21.43)	

AO: Acridine Orange, SDF: Sperm DNA Fragmentation.

**Table 3:** Correlations Between SDF and Clinical Parameters Using TUNEL and AO Methods.

	TUNEL		AO	
	R	*p*	R	*p*
Age (years)	0.07	0.59	0.311	0.02 *
Sperm parameters				
Initial concentration (M/mL)	0.029	0.842	−0.004	0.975
Progressive motility (%) ICSI parameters	−0.120	0.417	−0.08	0.527
Fertilization rate (%)	0.075	0.628	−0.08	0.545
Cleavage rate (%)	0.273	0.107	−0.226	0.161
Top Embryo rate (%)	0.01	0.919	−0.03	0.833
Blastulation rate (%)	−0.06	0.706	0.04	0.781
Blastocyst utilization rate (%)	0.008	0.970	0.267	0.139

This table presents the correlation coefficients (R) and *p*-values between SDF assessed by TUNEL and AO methods, and various clinical parameters. Parameters include age, sperm concentration, progressive motility, and ICSI outcomes such as fertilization rate, cleavage rate, top embryo rate, blastulation rate, and blastocyst utilization rate. The asterisk (*) indicates statistically significant results with a *p*-value of less than 0.05.

**Table 4:** Comparison Between Three Groups of SDF: Age, Semen and ICSI Parameters.

	SDF < 20				SDF > 20			
	TUNEL (*n* = 35)		AO (*n* = 44)		TUNEL (*n* = 20)		AO (*n* = 16)	
	R	*p*	R	*p*	R	*p*	R	*p*
Age (year)	0.226	0.313	0.04	0.801	−0.336	0.159	−0.191	0.463
Sperm concentration (M/mL)	0.01	0.946	−0.151	0.353	0.249	0.303	−0.240	0.353
Progressive motility (%)	0.005	0.982	−0.165	0.330	−0.02	0.940	−0.397	0.127
Number of MII oocytes	−0.152	0.499	0.148	0.435	−0.02	0.928	−0.05	0.872
ICSI parameters								
Fertilization Rate (%)	0.318	0.149	−0.100	0.569	−0.330	0.249	0.163	0.546
Cleavage rate (%)	0.241	0.335	−0.104	0.604	−0.360	0.250	−0.144	0.624
Top Embryo rate (%)	0.389	0.09	−0.05	0.762	0.01	0.959	0.485	0.07
Blastulation rate (%)	0.291	0.258	0.283	0.152	−0.160	0.602	0.424	0.149
Blastocyst utilization rate (%)	0.221	0.448	0.216	0.334	−0.247	0.555	0.424	0.181

This table compares the correlations (R) and *p*-values between clinical parameters and SDF assessed by TUNEL and AO methods, stratified by SDF levels (SDF < 20% and SDF > 20%). Parameters include age, sperm concentration, progressive motility, number of MII oocytes, and ICSI outcomes such as fertilization rate, cleavage rate, top embryo rate, blastulation rate, and blastocyst utilization rate.

## Data Availability

All data generated or analyzed during this study are included in this published article.

## Supplementary Materials

The following supporting information can be downloaded at https://www.mdpi.com/article/10.3390/jcm15020403/s1, Figure S1: Flow cytometry acquisition and gating strategy for the Acridine Orange.

## Abbreviations

The following abbreviations are used in this manuscript:

ISNT	In Situ Nick Translation
SSBs	Single-Strand Breaks
DSBs	Double-Strand Breaks
DNA	Deoxyribonucleic Acid
TdT	Terminal deoxynucleotidyl Transferase
PI	Propidium Iodide
β-hCG	Beta-Human Chorionic Gonadotropin
NaCl	Sodium Chloride
Tris	Tris(hydroxymethyl)aminomethane
EDTA	Ethylenediaminetetraacetic Acid
TNE	Tris-NaCl-EDTA Buffer
HCl	Hydrochloric Acid
Na_2_HPO_4_	Disodium Hydrogen Phosphate
PBS	Phosphate-Buffered Saline
SpermGrad™	Sperm Density Gradient Medium
GIVF™PLUS	Gamete Intrafallopian Transfer Medium Pl
FACS	Fluorescence-Activated Cell Sorting
SD	Standard Deviation
r	Correlation Coefficient
*p*	Probability Value (*p*-value)

## References

[1] Njagi P., Groot W., Arsenijevic J., Dyer S., Mburu G., Kiarie J. (2023). Financial Costs of Assisted Reproductive Technology for Patients in Low- and Middle-Income Countries: A Systematic Review. Hum. Reprod. Open.

[2] Sharma R.S., Saxena R., Singh R. (2018). Infertility & Assisted Reproduction: A Historical & Modern Scientific Perspective. Indian J. Med. Res..

[3] Yao D.F., Mills J.N. (2016). Male Infertility: Lifestyle Factors and Holistic, Complementary, and Alternative Therapies. Asian J. Androl..

[4] Sellami A., Chakroun N., Ben Zarrouk S., Sellami H., Kebaili S., Rebai T., Keskes L. (2013). Assessment of Chromatin Maturity in Human Spermatozoa: Useful Aniline Blue Assay for Routine Diagnosis of Male Infertility. Adv. Urol..

[5] Sengupta P., Dutta S., Samrot A.V., Singh R. (2023). Sperm DNA Fragmentation Testing in Infertility. Genetic Testing in Reproductive Medicine.

[6] Chatzimeletiou K., Fleva A., Nikolopoulos T.-T., Markopoulou M., Zervakakou G., Papanikolaou K., Anifandis G., Gianakou A., Grimbizis G. (2023). Evaluation of Sperm DNA Fragmentation Using Two Different Methods: TUNEL via Fluorescence Microscopy, and Flow Cytometry. Medicina.

[7] Sharma R., Iovine C., Agarwal A., Henkel R. (2021). TUNEL Assay—Standardized Method for Testing Sperm DNA Fragmentation. Andrologia.

[8] Chohan K.R., Griffin J.T., Lafromboise M., De Jonge C.J., Carrell D.T. (2006). Comparison of Chromatin Assays for DNA Fragmentation Evaluation in Human Sperm. J. Androl..

[9] Evgeni E., Charalabopoulos K., Asimakopoulos B. (2014). Human Sperm DNA Fragmentation and Its Correlation with Conventional Semen Parameters. J. Reprod. Infertil..

[10] Evgeni E., Sabbaghian M., Saleh R., Gül M., Vogiatzi P., Durairajanayagam D., Jindal S., Parmegiani L., Boitrelle F., Colpi G. (2023). Sperm DNA Fragmentation Test: Usefulness in Assessing Male Fertility and Assisted Reproductive Technology Outcomes. Panminerva Medica.

[11] Henkel R., Hajimohammad M., Stalf T., Hoogendijk C., Mehnert C., Menkveld R., Gips H., Schill W.-B., Kruger T.F. (2004). Influence of Deoxyribonucleic Acid Damage on Fertilization and Pregnancy. Fertil. Steril..

[12] Hamidi J., Frainais C., Amar E., Bailly E., Clément P., Ménézo Y. (2015). A Double-Blinded Comparison of in Situ TUNEL and Aniline Blue versus Flow Cytometry Acridine Orange for the Determination of Sperm DNA Fragmentation and Nucleus Decondensation State Index. Zygote.

[13] Sun T.-C., Zhang Y., Li H.-T., Liu X.-M., Yi D.-X., Tian L., Liu Y.-X. (2018). Sperm DNA Fragmentation Index, as Measured by Sperm Chromatin Dispersion, Might Not Predict Assisted Reproductive Outcome. Taiwan. J. Obstet. Gynecol..

[14] Ribas-Maynou J., Yeste M., Becerra-Tomás N., Aston K.I., James E.R., Salas-Huetos A. (2021). Clinical Implications of Sperm DNA Damage in IVF and ICSI: Updated Systematic Review and Meta-Analysis. Biol. Rev..

[15] Evenson D.P., Thompson L., Jost L. (1994). Flow Cytometric Evaluation of Boar Semen by the Sperm Chromatin Structure Assay as Related to Cryopreservation and Fertility. Theriogenology.

[16] Santi D., Spaggiari G., Simoni M. (2018). Sperm DNA Fragmentation Index as a Promising Predictive Tool for Male Infertility Diagnosis and Treatment Management—Meta-Analyses. Reprod. Biomed. Online.

[17] Cortés-Gutiérrez E.I., Fernández J.L., Dávila-Rodríguez M.I., López-Fernández C., Gosálvez J., Pellicciari C., Biggiogera M. (2017). Two-Tailed Comet Assay (2T-Comet): Simultaneous Detection of DNA Single and Double Strand Breaks. Histochemistry of Single Molecules: Methods and Protocols.

[18] Esteves S.C., Zini A., Coward R.M., Evenson D.P., Gosálvez J., Lewis S.E.M., Sharma R., Humaidan P. (2021). Sperm DNA Fragmentation Testing: Summary Evidence and Clinical Practice Recommendations. Andrologia.

[19] Agarwal A., Farkouh A., Saleh R., Hamoda T.A.A.A.M., Salvio G., Boitrelle F., Harraz A.M., Ghayda R.A., Kavoussi P., Gül M. (2024). Technical Aspects and Clinical Limitations of Sperm DNA Fragmentation Testing in Male Infertility: A Global Survey, Current Guidelines, and Expert Recommendations. World J. Mens. Health.

[20] Gosálvez J., López-Fernández C., Fernández J., Esteves S., Johnston S. (2015). Unpacking the Mysteries of Sperm DNA Fragmentation: Ten Frequently Asked Questions. J. Reprod. Biotechnol. Fertil..

[21] Adler A., Roth B., Lundy S.D., Takeshima T., Yumura Y., Kuroda S. (2023). Sperm DNA Fragmentation Testing in Clinical Management of Reproductive Medicine. Reprod. Med. Biol..

[22] Rhouma M.B., Bahri H., Khalifa M.B., Sakly M., Rhouma K.B., Benkhalifa M., Tebourbi O. (2025). Oxidative Stress and Its Correlation with Sperm Parameters in Different Semen Quality Groups. Clin. Exp. Reprod. Med..

[23] Yao G., Dou X., Chen X., Qi H., Chen J., Wu P., Li J., Liang S., Han Z., Bai S. (2024). Association between Sperm DNA Fragmentation Index and Recurrent Pregnancy Loss: Results from 1485 Participants Undergoing Fertility Evaluation. Front. Endocrinol..

[24] Samli M., Samli H., Gul C.B., Ersoy A., Ardicli S., Balci F. (2022). TUNEL Analysis of Sperm DNA Fragmentation in Kidney Transplant Patients. J. Cell. Biotechnol..

[25] Dutta S., Henkel R., Agarwal A. (2021). Comparative Analysis of Tests Used to Assess Sperm Chromatin Integrity and DNA Fragmentation. Andrologia.

[26] Elango K., Kumaresan A., Talluri T., Raval K., Paul N., Ebenezer Samuel King J.P., Sinha M., Patil S. (2022). Impact of Sperm Protamine on Semen Quality and Fertility. J. Reprod. Healthc. Med..

[27] Simon L., Castillo J., Oliva R., Lewis S.E.M. (2011). Relationships between Human Sperm Protamines, DNA Damage and Assisted Reproduction Outcomes. Reprod. Biomed. Online.

[28] Esteves S.C., Sharma R.K., Gosálvez J., Agarwal A. (2014). A Translational Medicine Appraisal of Specialized Andrology Testing in Unexplained Male Infertility. Int. Urol. Nephrol..

[29] Yang B., Xia L., Deng R., Wu L., Zhang Z., Wu X., Ding T., Zhao Y., Huang J., Huang Z. (2025). Impact of Sperm DNA Fragmentation Index on Assisted Reproductive Outcomes: A Retrospective Analysis. Front. Endocrinol..

[30] Agarwal A., Baskaran S., Parekh N., Cho C.-L., Henkel R., Vij S., Arafa M., Panner Selvam M.K., Shah R. (2021). Male Infertility. Lancet.

[31] Colaco S., Sakkas D. (2018). Paternal Factors Contributing to Embryo Quality. J. Assist. Reprod. Genet..

[32] Zribi N., Chakroun N.F., Elleuch H., Abdallah F.B., Ben Hamida A.S., Gargouri J., Fakhfakh F., Keskes L.A. (2011). Sperm DNA Fragmentation and Oxidation Are Independent of Malondialdheyde. Reprod. Biol. Endocrinol..

[33] Gong P., Guo Z., Wang S., Gao S., Cao Q. (2025). Histone Phosphorylation in DNA Damage Response. Int. J. Mol. Sci..

[34] Cohen-Bacrie P., Belloc S., Ménézo Y.J.R., Clement P., Hamidi J., Benkhalifa M. (2009). Correlation between DNA Damage and Sperm Parameters: A Prospective Study of 1,633 Patients. Fertil. Steril..

[35] Henkel R., Hoogendijk C.F., Bouic P.J.D., Kruger T.F. (2010). TUNEL Assay and SCSA Determine Different Aspects of Sperm DNA Damage. Andrologia.

[36] Osman A., Alsomait H., Seshadri S., El-Toukhy T., Khalaf Y. (2015). The Effect of Sperm DNA Fragmentation on Live Birth Rate after IVF or ICSI: A Systematic Review and Meta-Analysis. Reprod. Biomed. Online.

[37] Wu T.F., Chu D.S. (2008). Sperm Chromatin: Fertile Grounds for Proteomic Discovery of Clinical Tools. Mol. Cell. Proteom..

[38] Vahedi Raad M., Firouzabadi A.M., Tofighi Niaki M., Henkel R., Fesahat F. (2024). The Impact of Mitochondrial Impairments on Sperm Function and Male Fertility: A Systematic Review. Reprod. Biol. Endocrinol..

[39] Condorelli R.A., La Vignera S., Bellanca S., Vicari E., Calogero A.E. (2012). Myoinositol: Does It Improve Sperm Mitochondrial Function and Sperm Motility?. Urology.

[40] Zhang H., Li Y., Wang H., Zhou W., Zheng Y., Ye D. (2022). Does Sperm DNA Fragmentation Affect Clinical Outcomes during Vitrified-Warmed Single-Blastocyst Transfer Cycles? A Retrospective Analysis of 2034 Vitrified-Warmed Single-Blastocyst Transfer Cycles. J. Assist. Reprod. Genet..

[41] Fu W., Cui Q., Yang Z., Bu Z., Shi H., Bi B., Yang Q., Xin H., Shi S., Hu L. (2023). High Sperm DNA Fragmentation Increased Embryo Aneuploidy Rate in Patients Undergoing Preimplantation Genetic Testing. Reprod. Biomed. Online.

[42] Chua S.C., Yovich S.J., Hinchliffe P.M., Yovich J.L. (2023). The Sperm DNA Fragmentation Assay with SDF Level Less Than 15% Provides a Useful Prediction for Clinical Pregnancy and Live Birth for Women Aged under 40 Years. J. Pers. Med..

