# Supplementary material for: Flow Cytometric Assessment of Sperm DNA Fragmentation by TUNEL and Acridine Orange: Methodological and Clinical Insights
Source: J Clin Med. 2026 Jan 6;15(2):403. doi: 10.3390/jcm15020403 (PMC12842179; doi:10.3390/jcm15020403)
Supplement: Supplementary file 1 [file jcm-15-00403-s001.zip › jcm-3976097-supplementary.pdf]

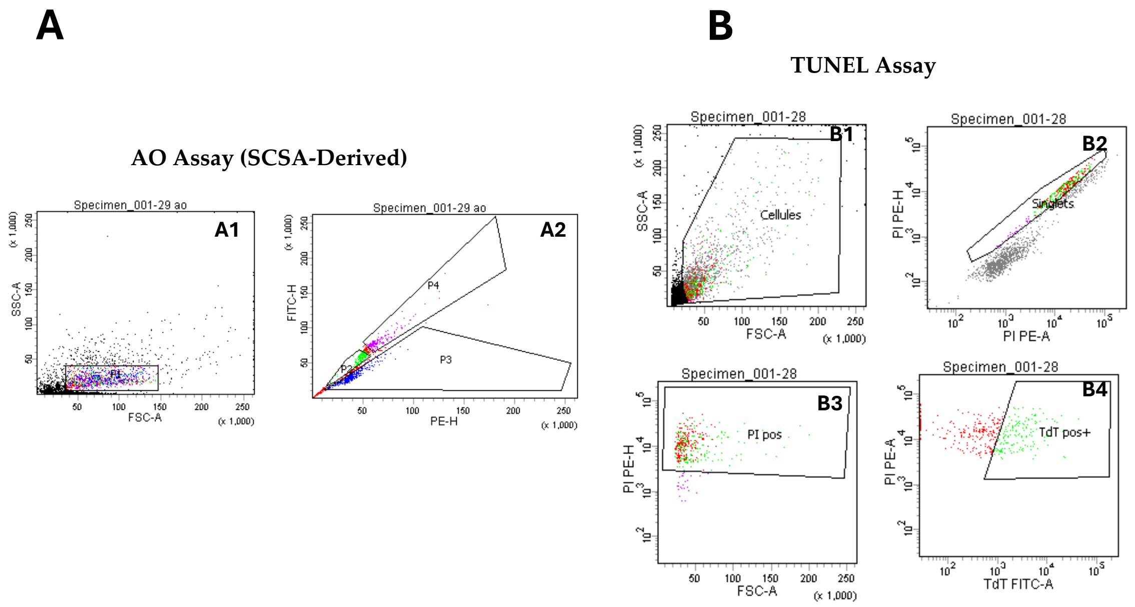

Figure S1. Flow cytometry acquisition and gating strategy for the Acridine Orange (A) and TUNEL (B) assays.

(A) Acridine Orange (AO) assay.
(A1) *FSC-A vs. SSC-A plot*: the main sperm population was identified, and debris were excluded.
(A2) *AO metachromatic fluorescence plot*: green fluorescence (FL1) corresponds to intact double-stranded DNA, whereas red fluorescence (FL3) indicates denatured or single-stranded DNA. The red-fluorescent population represents the metachromatic fluorescence distribution (MFD) and is used to calculate SDF (%)**.**

(B) TUNEL assay.
(B1) *FSC-A vs. SSC-A*: gating of the sperm population after removal of debris.
(B2) *PI-A vs. PI-H plot*: selection of singlets to exclude doublets and aggregates.
(B3) *TUNEL fluorescence plot (FITC vs. PI)*: FITC-positive spermatozoa represent fragmented DNA following incorporation of fluorescein-dUTP.
(B4) *Positive control*: the TdT-positive sample shows the expected shift toward the FITC-positive region and was used to define the threshold for TUNEL-positive events.
